# Diagnostic Dilemma: Unraveling Meige Disorder Mistaken for Functional Neurological Disorder

**DOI:** 10.7759/cureus.61465

**Published:** 2024-05-31

**Authors:** Yatika Chadha, Saket Toshniwal, Ragini Patil

**Affiliations:** 1 Psychiatry, Datta Meghe Institute of Higher Education and Research, Jawaharlal Nehru Medical College, Wardha, IND; 2 General Medicine, Datta Meghe Institute of Higher Education and Research, Jawaharlal Nehru Medical College, Wardha, IND

**Keywords:** functional neurological disorder, stigma, diagnostic challenges, misdiagnosis, meige syndrome

## Abstract

Meige syndrome, a rare form of cranial dystonia, manifests as involuntary spasms affecting the facial and neck muscles. Diagnosing Meige syndrome is challenging due to its similarities with various movement disorders and psychiatric conditions. Functional neurological disorder (FND) refers to a condition characterized by neurological symptoms that are inconsistent with recognized neurological or medical conditions. Symptoms may include motor or sensory disturbances such as weakness, tremors, paralysis, or seizures. Importantly, these symptoms cannot be fully explained by another medical condition or by the direct effects of a substance. Instead, they are believed to stem from psychological factors. This case demonstrates the diagnostic dilemma of Meige syndrome. It was initially misdiagnosed as a functional neurological disorder in a 42-year-old female. The difficulties in differentiating between these disorders highlight the necessity of a thorough evaluation and increased clinical suspicion in cases of movement disorders. For treatment outcomes to be optimized and to resolve patient distress, prompt and accurate diagnosis is essential.

## Introduction

Meige disorder, also known as oromandibular dystonia, is a rare neurological movement disorder characterized by involuntary, repetitive contractions of the muscles of the face, mouth, and jaw. It was named after the French neurologist Henri Meige, who first described it in 1910. This condition manifests as involuntary spasms, grimacing and abnormal movements affecting the oromandibular region, often leading to significant functional impairment and distress for those afflicted [1].

While precise prevalence figures for Meige disorder remain elusive due to its rarity and under-diagnosis, it is estimated to affect approximately one to nine individuals per million in the general population [2]. However, this figure may be an underestimation given the challenges in diagnosis and the lack of awareness among healthcare professionals.

The pathophysiology underlying Meige disorder is complex and multifactorial, involving dysfunction within the basal ganglia-thalamus and cortical motor circuitry [3]. Emerging evidence suggests that abnormalities in neurotransmitter systems, particularly involving gamma-aminobutyric acid (GABA) and dopamine, contribute to the development and progression of dystonic symptoms. Structural abnormalities within the basal ganglia, alterations in cortical excitability and aberrant plasticity mechanisms further exacerbate motor dysfunction in affected individuals.

Understanding the underlying pathophysiology of Meige disorder is crucial for the development of targeted therapeutic interventions and treatment protocols aimed at alleviating symptoms and improving quality of life that is health-related quality of life (HR-QoL) for patients [4].

Functional neurological disorder (FND), also known as conversion disorder, is a condition characterized by neurological symptoms that cannot be attributed to an underlying medical or neurological condition. These symptoms often mimic those of neurological disorders, such as weakness, paralysis, tremors, or seizures, but lack a clear organic cause. Instead, they are believed to arise from psychological factors, such as stress, trauma, or emotional distress [5].

In this case report, we present a comprehensive analysis of a patient diagnosed with Meige disorder, earlier misdiagnosed as a functional neurological disorder, highlighting the diagnostic challenges, therapeutic interventions, clinical presentation and outcomes, with reference to the current literature and emerging research in the field.

## Case presentation

A 42-year-old married female was brought to the psychiatry outpatient department after being referred from the medicine and otolaryngology departments with complaints of difficulty speaking due to twitching in facial muscles, involuntary twitching of eyelids (blepharospasm), and spasms of neck muscles since the past four years. The patient also complained of difficulty in swallowing semisolids and liquids. Further evaluation revealed depressive features in the patient, including persistent low mood, decreased interest in daily activities, decreased interaction with others, decreased sleep, and anxiety. A general physical examination of the patient revealed a pulse rate of 80 beats per minute of normal rhythm and volume measured in the right radial artery, a blood pressure of 120/80 mmHg measured in the supine position in the right arm, a respiratory rate of 18 cycles per minute, and an oxygen saturation of 98% on room air. The patient showed no signs of pallor, icterus, edema, cyanosis, clubbing, or lymphadenopathy. The system examination of the patient was unremarkable with neurological examination revealing oromandibular, facial and cervical dystonic movements with dysarthria and dysphagia. The sensory and cerebellar examinations were unremarkable, revealing normal deep tendon and superficial reflexes, as well as normal power and tone in all four limbs during the motor examination. Table 1 shows the results of all routine blood investigations, including all serum electrolytes, thyroid profile, and lipid profile, which were all within normal limits.

**Table 1:** All routine blood investigations of the patient.

Investigation	Normal range	Results
Blood glucose	Fasting: 70-100 mg/dL	90 mg/dL
White blood cells	4,500-11,000 cells/mm³	7,000 cells/mm³
Triglycerides	<150 mg/dL	100 mg/dL
Total cholesterol	<200 mg/dL	180 mg/dL
Total bilirubin	0.1-1.2 mg/dL	0.8 mg/dL
Thyroid-stimulating hormone (TSH)	0.4-4.0 mIU/L	2.0 mIU/L
Sodium (Na)	135-145 mmol/L	140 mmol/L
Serum creatinine (SCr)	0.6-1.3 mg/dL	0.9 mg/dL
Potassium (K)	3.5-5.1 mmol/L	4.2 mmol/L
Platelets	150,000-400,000/mm³	250,000/mm³
LDL cholesterol	<100 mg/dL	70 mg/dL
Hemoglobin (Hb)	13.8-17.2 g/dL	15.0 g/dL
Hematocrit (Hct)	40-54%	45%
HDL cholesterol	>40 mg/dL	50 mg/dL
Free triiodothyronine (FT3)	2.3-4.2 pg/mL	3.8 pg/mL
Free thyroxine (FT4)	0.8-2.0 ng/dL	1.5 ng/dL
Chloride (Cl)	98-106 mmol/L	102 mmol/L
Calcium (Ca)	8.5-10.5 mg/dL	9.2 mg/dL
Blood urea nitrogen (BUN)	7-20 mg/dL	15 mg/dL
Aspartate aminotransferase (AST)	10-30 U/L	25 U/L
Alkaline phosphatase (ALP)	20-140 U/L	70 U/L
Alanine aminotransferase (ALT)	10-40 U/L	20 U/L

**Figure 1:**
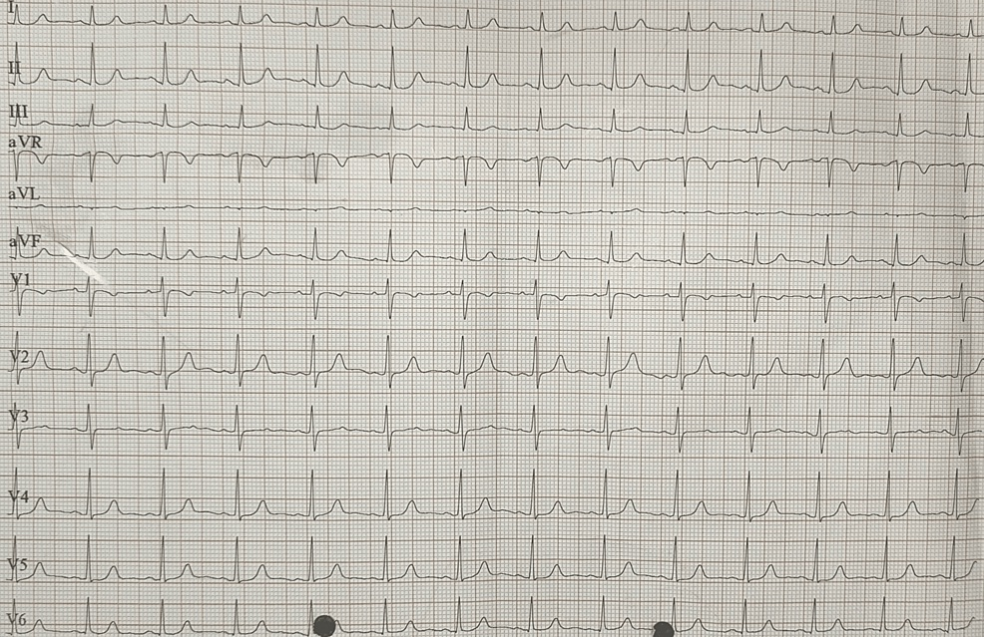
Electrocardiogram of the patient showing normal sinus rhythm.

An electrocardiogram was conducted, which was suggestive of normal sinus rhythm (Figure 1).

**Figure 2:**
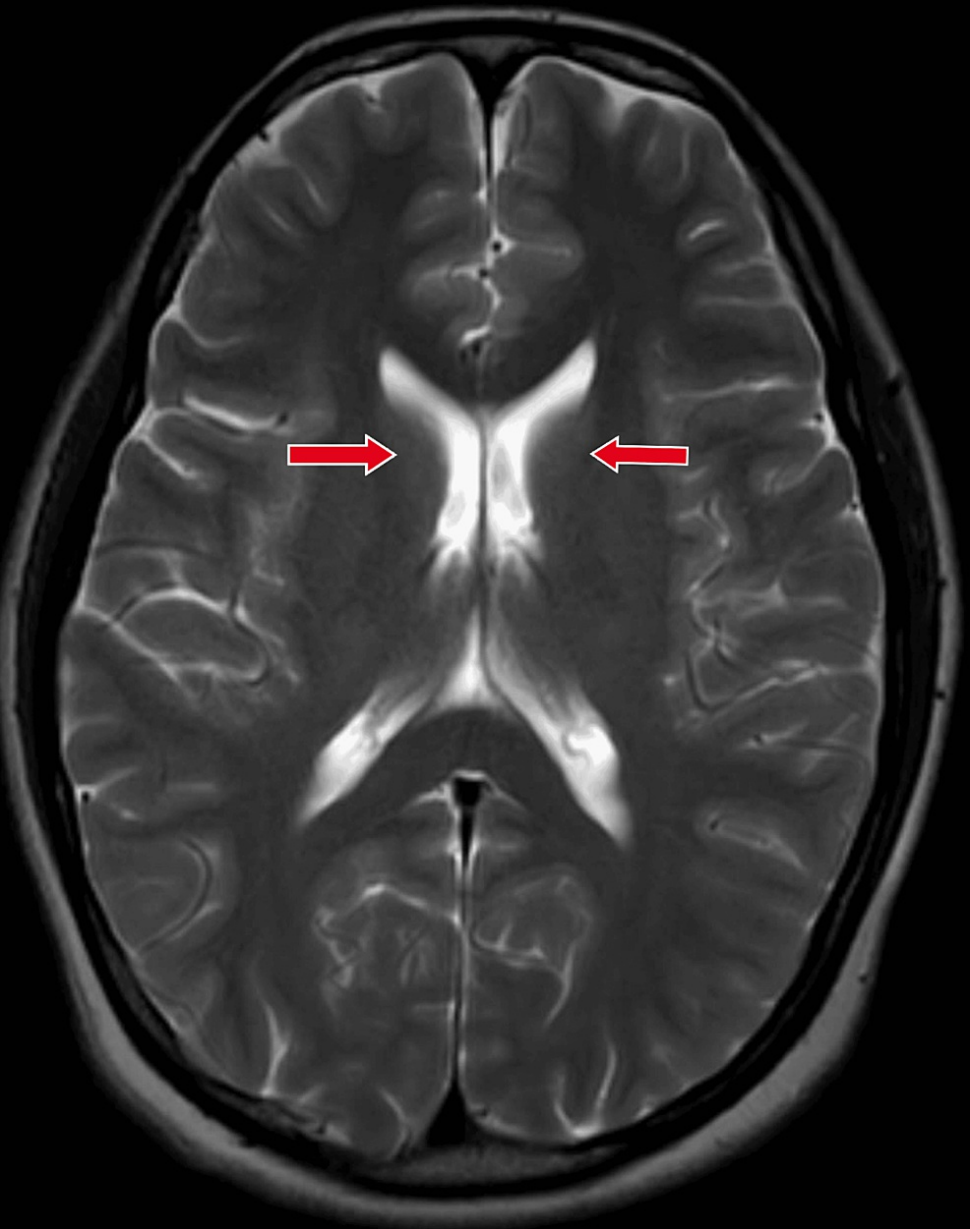
MRI T2-weighted axial section of brain suggesting normal imaging study. Magnetic resonance imaging (MRI) of the brain with normal appearing non-atrophied bilateral caudate nucleus with no abnormal hypo- or hyper-intensities shown with red arrows.

A magnetic resonance imaging (MRI) brain scan was done in order to rule out any organic causes for the presenting symptoms and was found to be normal (Figure 2).

She underwent a video laryngoscopy to rule out any organic basis for her dysarthria, and the results showed that her vocal cords were anatomically and functionally normal. A gastroscopy was performed considering her dysphagia to rule out any organic cause, which showed no abnormalities. Conditions like stroke, neoplasms (meningioma or a metastatic tumor), autoimmune diseases like multiple sclerosis and lupus, and inherited disorders like Wilson’s disease were also ruled out, owing to the normal magnetic resonance imaging of her brain. The patient was first started on tablet escitalopram 10 mg and tablet clonazepam 0.25 mg once at night with a provisional diagnosis of functional neurological disorder after all other organic differentials were ruled out. Following up revealed an inadequate response; therefore, a trial of a 2.5 mg tablet of olanzapine and a 15 mg dose of escitalopram was administered; however, this too did not result in any improvement. On subsequent follow-ups, tablet baclofen 5 mg was started with tablet clonazepam 0.25 mg once at night with a continuation of escitalopram, tablet olanzapine was stopped, and the diagnosis of functional neurological disorder was revised to oromandibular dystonia under evaluation. At the subsequent follow-up, her dysphagia and persistent dysarthria symptoms remained unabated. Consequently, a neurology opinion was obtained, where she was diagnosed with a case of oromandibular pharyngeal lingual dystonia and started on tablet syndopa plus 125 mg half tablet thrice a day (1/2-1/2-1/2) and tablet trihexyphenidyl 2 mg thrice a day along with tablet clonazepam 0.25 mg one tablet once at night. After two weeks, the patient's symptoms started to improve. For instance, she could now utter a few phrases, and her involuntary muscle movements were progressing at a lower intensity. Her depressive symptoms were also getting better. The patient's dystonic movements continued, nevertheless. She was then closely monitored by the departments of neurology as well as psychiatry. The patient was counseled regarding her condition; she was made to understand the complexity and the long course of the illness. Her relatives were sensitized regarding her condition and the debilitating effects of the illness on her mental health primarily and the need for regular follow-ups.

## Discussion

This was a case of a pronounced presentation of Meige syndrome with the patient experiencing chewing and speaking difficulties, where multiple follow-ups to doctors of every specialty and multiple investigations were done to rule out causes for the illness, and then, in the end, she was referred to a psychiatrist, where she was misdiagnosed as a case of functional neurological disorder, and then the diagnosis was revised to Meige syndrome with secondary depressive symptoms. This illness has been frequently misdiagnosed as a case of functional neurological disorder since time immemorial due to multiple factors [6].

In studies, a pattern of precipitating factors like emotional stress is associated, but it has not been seen consistently in the current evidence. Whereas a positive history of movement disorders, such as Parkinson’s disease, has been noted in a few studies [7]. This disorder was seen primarily in females around the world, in their late 50s, some studies show the younger population being affected by it too [4]. The treatment entailed multimodalities with adequate psychotherapies and pharmacotherapies. Prior studies have shown effective responses to monotherapy of tetrabenazine 75 mg [8]. However, maximum evidence and effectiveness have been seen for combination therapy with clonazepam, trihexyphenidyl, and baclofen. Studies have also shown the role of gamma-aminobutyric acid (GABA) agonists in skeletal muscle relaxants like baclofen and atypical antipsychotics like clozapine and tetrabenazine [6,8,9]. Here, in our case, the patient was started on escitalopram and baclofen and later was put on syndopa 125 mg with an anticholinergic, which worked efficaciously to improve her symptoms. 

Although the exact pathophysiological mechanism of Meige syndrome is not fully understood, it is believed to involve basal ganglia-thalamocortical motor circuitry disturbances, which play a crucial role in the regulation of voluntary motor movements. Studies suggest that abnormalities in neurotransmitter systems, particularly dopamine and gamma-aminobutyric acid (GABA), may further contribute to the development of dystonic symptoms [10]. Dopamine, a neurotransmitter involved in the modulation of motor control and coordination is mainly associated with the pathogenesis of dystonia. Dysfunction in dopaminergic signaling pathways, particularly in the striatum, may disturb the balance between excitatory and inhibitory signals within the basal ganglia, leading to abnormal muscle contractions and involuntary movements, hence causing dystonic movements [10]. Additionally, alterations in GABAergic neurotransmission, which serves as the primary inhibitory neurotransmitter in the central nervous system, have also been associated with the pathophysiology of dystonia by impairing motor inhibition due to dysfunction of circuits between basal ganglia and cortex [10]. 

Before diagnosing Meige syndrome, various close differential diagnoses like Parkinson's disease, tardive dyskinesia, hemifacial spasm, Wilson's disease, primary cranial dystonia, and various secondary causes of dystonia such as stroke, traumatic brain injury, multiple sclerosis, or certain metabolic disorders, which present similar to Meige syndrome should be ruled out. A comprehensive medical history, neurological examination and appropriate investigations like imaging studies help rule out these differentials [11].

The lack of awareness about the illness among the general population, along with the debilitating effects due to depression, leads to a misdiagnosis of this case as a functional neurological disorder [12]. After going through previous research that had similar diagnostic conundrums, the presence of secondary gain with the presenting state and the degree of indifference towards the symptoms or the massive denial that is seen in hysteria, which was not present in our case, were noted as indicators against a diagnosis that could help in differentiating functional neurological disorder from Meige's disorder [10]. The burden of the illness on the patient, which included difficulty in chewing or speaking, added to the social stigmas and psychological stress along with declining quality of life, making it necessary to have a high index of suspicion to prevent future misdiagnosis [13]. 

Further research is needed to elucidate the underlying mechanisms driving dystonic symptoms in order to develop more effective therapeutic strategies. Moreover, such reports are important to be brought to literature to create awareness among the practitioners to consider the possibility of Meige syndrome as a differential to help early diagnose and promptly treat this rare condition.

## Conclusions

The case study concludes the need to identify Meige syndrome and emphasizes the need for rigorous evaluation and cautious thought in situations when movement disorders manifest. The initial mistake of dissociative disorder, like in our case, serves as a warning about potential diagnostic problems and emphasizes the need for thorough examination methodologies and the maintenance of a broad differential diagnosis. These diagnostic challenges can be reduced in healthcare workers by raising awareness, which would allow prompt identification and proper management of Meige syndrome. Comprehensive healthcare policies that prioritize early detection, provide equitable access to treatment alternatives, and encourage interdisciplinary collaboration among healthcare professionals require collaborative efforts to design. Moreover, funding public health campaigns to increase knowledge about dystonia illnesses, such as Meige syndrome, can lessen stigma, encourage early intervention, and enhance results for those who are affected. Policymakers can help create a more inclusive and supportive healthcare system that enables people with Meige syndrome to live a happy and meaningful lives by putting the needs of patients and their families first.

Once the complexity of Meige syndrome is resolved, we may be able to improve patient outcomes and elevate the standard of care for people suffering from this challenging condition, avoiding further misdiagnosis and prolonged management. 
